# The Role of Diet and Gut Microbiome in CKD Progression and Therapy

**DOI:** 10.3390/jcm15103934

**Published:** 2026-05-20

**Authors:** Wei Ling Lau, Whitney Li, Keiichi Sumida, Kamyar Kalantar-Zadeh

**Affiliations:** 1 Division of Nephrology, Department of Medicine, University of California-Irvine, Irvine, CA 92697, USA; wllau@uci.edu (W.L.L.);; 2 Division of Nephrology, Department of Medicine, University of Tennessee Health Science Center, Memphis, TN 38163, USA; 3 Nephrology Section, Veterans Affairs Greater Los Angeles Healthcare System, Los Angeles, CA 90073, USA; 4 David Geffen School of Medicine, University of California Los Angeles, Los Angeles, CA 90095, USA; 5 Lundquist Biomedical Research Institute at Harbor-UCLA Medical Center, Torrance, CA 90502, USA

**Keywords:** diet, gut microbiome, uremic toxins, chronic kidney disease

## Abstract

There is a bidirectional relationship between chronic kidney disease (CKD) and an altered gut microbiome, with gut-derived uremic toxins contributing to cardiovascular-kidney-metabolic effects. In this review, we summarize the interplay between diet, the intestinal microbiota and systemic sequelae including CKD progression, cardiovascular morbidity and cognitive decline. We discuss the current state of knowledge regarding microbiota-modulating therapies that have the potential to delay CKD complications such as plant-dominant diets, oral adsorbents, prebiotics/probiotics, fecal microbiota transplantation and exercise.

## 1. Introduction

Chronic kidney disease (CKD) is a global health issue leading to multi-organ complications including heightened cardiovascular morbidity. In 2023, the global CKD prevalence was 14.2% among adults aged 20 years and older, and CKD was the 9th leading cause of death [1] (having previously been the 17th leading cause of death worldwide in 1990 [2]). CKD is recognized as an independent risk factor for cardiovascular morbidity even after adjustment for traditional cardiovascular risk factors, with the prevalence of cardiovascular disease being 75.3% in stage 4 CKD as compared to 37.5% in the non-CKD population [3]. In 2023, 11.5% of global deaths due to cardiovascular disease were attributable to CKD [1]. Non-traditional risk factors contributing to cardiovascular disease in CKD patients include disrupted mineral–bone metabolism, chronic inflammation and oxidative stress, a deficiency of inhibitors of vascular calcification inhibitors, and protein–energy wasting [4]. There is growing evidence that metabolites from the gut microbiota promote this cardiovascular risk. Advances in microbial sequencing have revealed a bidirectional relationship between CKD and the intestinal flora, with dysbiosis and gut-derived toxin levels correlating with CKD complications, including vascular disease, renal fibrosis, anemia, and osteodystrophy [4,5,6]. This review article aims to summarize the interplay between diet, the intestinal microbiota and CKD, and discuss the potential of gut-targeted therapies to delay CKD complications.

## 2. Bidirectional Kidney-Gut Axis

### 2.1. Gut Microbial Changes in CKD

The human gut microbiota is a diverse ecological system that includes bacteria, yeast, viruses and parasites, yielding around 100 trillion microorganisms in total [7,8,9,10]. At birth, the sterile human gut is rapidly colonized by the mother’s microbiome, which helps to shape the infant’s immune system [11]. The healthy gut microbiota is predominantly represented by the phyla *Firmicutes* and *Bacteroidetes* (90% of the gut flora); *Firmicutes* includes several genera, of which the most common are *Lactobacillus*, *Bacillus*, *Enterococcus*, *Ruminicoccus* and *Clostridium* [12]. The normal microbial flora has critical roles in host health: shaping the adaptive immune system after birth, regulating local and systemic immunity, producing micronutrients for colonocyte health, including short-chain fatty acids (SCFAs) and vitamins (group B vitamins and vitamin K), and contributing to nitrogen balance (synthesis of amino acids such as lysine and threonine) [4].

In CKD, retained toxins, including urea accumulate and diffuse from the blood into the gut lumen, stimulating the proliferation of bacteria that express urease and altering the gut microbial population [5,13]. Luminal urea is converted to ammonia and subsequently to caustic ammonium hydroxide, which disrupts the epithelial barrier, leading to the translocation of endotoxin and gut microbial products (including uremic toxins) into the blood circulation. Additionally, patients with advanced CKD are often advised to adhere to a diet that is low in potassium and phosphate, which often translates to a decreased intake of vegetables, dairy and yogurt—this deprives the diet of fiber and symbionts which further alters the gut microbiota [4,14]. When there is adequate fiber intake, plant-derived resistant starches transit intact to the colon, where they are degraded by Bacteroides and fermented to release SCFAs (including acetate, butyrate, propionate and D-lactate), which are important nutrients for colonocytes [15]. The combined altered gut microbiota concurrent with the depletion of fiber in the CKD diet decreases SCFA generation, which detrimentally impacts the health of colon epithelial cells. Fecal analysis has confirmed that dialysis patients show decreased numbers of bacteria that are able to produce SCFAs [16]. The most frequently reported gut microbiota changes in CKD are related to decreased Bifidobacteriaceae and Lactobacillaceae (the latter being important SCFAs producers) and increased levels of Enterobacteriaceae [6]. In stool microbial sequencing from end-stage kidney disease (ESKD) patients, compared with age-, sex- and ethnicity-matched healthy individuals, there were significant differences in the abundance of over 200 bacterial species belonging to 23 bacterial families [17]. Microbial families showing the largest increase in ESKD patients belong to the Actinobacteria, *Firmicutes* (especially subphylum Clostridia), and *Proteobacteria* (primarily Gamma*Proteobacteria*) phyla [17] (Table 1). The gut microbial alterations lead to increased production of uremic toxins as discussed below.

### 2.2. Gut-Derived Uremic Toxins

Gut microbiota that become prominent in CKD, such as Clostridiaceae and Enterobacteriaceae possess urease, uricase, indole- and p-cresyl-forming enzymes [16,17]. As a result, there is a shift to a proteolytic phenotype whereby the microbes catabolize amino acids to generate gut-derived uremic metabolites, such as indoxyl sulfate, p-cresyl sulfate and trimethylamine-N-oxide (TMAO), which translocate across the gut wall into the bloodstream and incur systemic toxicity and inflammation. Gut-derived toxins are categorized as protein-bound vs water-soluble [4,19]. Indoxyl sulfate and indole-3 acetic acid are protein-bound uremic toxins that arise from tryptophan catabolism. Tryptophan is initially metabolized into indole by the gut microbiota, and after intestinal absorption is sulfated in the liver. P-cresyl sulfate is produced from phenylalanine and tyrosine catabolism by gut bacteria, and is metabolized in the liver to p-cresyl glucuronide. Indoxyl sulfate and p-cresyl sulfate are protein-bound and are not efficiently removed by dialysis. In contrast, TMAO is derived from the bacterial metabolism of quaternary amines, such as choline and L-carnitine, to trimethylamine, which is subsequently converted to water-soluble TMAO by flavin monooxygenase enzymes in the liver.

Dietary restrictions and medications that are routinely imposed on CKD patients further modulate the gut microbiota [4]. A decreased intake of fruits/vegetables to avoid hyperkalemia, and a reduced dairy/yogurt intake to limit hyperphosphatemia, respectively, lead to a low-fiber diet and decreased exposure to symbionts. Combined, these factors promote a proteolytic rather than saccharolytic microbiome. Common medications in CKD patients have variable effects on the microbiome, and may increase (proton pump inhibitors [20]) or decrease (phosphate binders [21], antibiotics [22]) uremic toxin generation, or exert mixed effects (iron supplements [23,24]).

### 2.3. Multi-Organ Effects of Gut-Derived Uremic Toxins

Circulating levels of gut microbiota-derived uremic toxins have been implicated in systemic end-organ injury, including CKD progression, cardiovascular disease, diabetes mellitus risk and cognitive decline. In terms of CKD progression, an elevated level of the gut metabolite TMAO alters pyruvate metabolism in renal tubular epithelial cells, with the upregulation of histone H4 lysine 12 lactylation driving macrophage M2 polarization and fibrosis [25]. Preclinical investigations demonstrate that indoxyl sulfate and p-cresyl sulfate stimulate monocyte inflammation and oxidative stress [26,27], with indoxyl sulfate additionally incurring podocyte toxicity and foot process effacement [28]. These processes culminate in the progression of kidney fibrosis and the loss of renal function. Further, gut-derived toxins in CKD have been implicated in the pathogenesis of anemia and bone osteodystrophy [4,5,6].

The majority of outcomes research in the field of uremic toxins has been related to cardiovascular events and mortality. Cardiovascular disease is prevalent in 50% of all patients with CKD stage 4 or higher, and cardiovascular mortality accounts for 40–50% of all deaths in this population compared with 26% in controls with normal kidney function [29,30]. CKD drives cardiovascular risk independent of classical factors, such as diabetes mellitus, hypertension, smoking and dyslipidemia, due to non-traditional risk factors including uremic toxins, mineral dysmetabolism and protein–energy wasting. Indoxyl sulfate is one example of a gut-derived toxin that exerts detrimental effects on the vasculature. In preclinical studies, indoxyl sulfate has been shown to impair endothelial proliferation and to induce endothelial-to-mesenchymal transition [31,32,33], promote cellular senescence [34,35], and stimulate vascular smooth muscle cell proliferation [36]. In CKD patient cohorts, indoxyl sulfate has been correlated with aortic calcification, heart failure, and cardiovascular mortality [37,38,39]. Dietary L-carnitine supplementation in mice has been shown to increase blood TMAO and to promote atherosclerosis; these effects were attenuated by treatment with broad-spectrum antibiotics to suppress the gut microbiota [40]. Plasma TMAO concentrations correlate with an increased mortality risk in the CKD population [41,42].

Gut-derived uremic toxins have been implicated in insulin resistance and the risk of diabetes mellitus. In vitro studies with adipocytes and myocytes treated with indoxyl sulfate or p-cresyl sulfate demonstrated an increased production of reactive oxygen species, concurrent with insulin resistance [43,44]; intraperitoneal injection of p-cresyl sulfate for 4 weeks in healthy mice induced insulin resistance, concurrent with the ectopic redistribution of lipid in the skeletal muscle and the liver [44]. Tongu and colleagues reported preclinical investigations demonstrating that the toxin phenyl sulfate increased pancreatic insulin secretion, which was opposed by adipocyte insulin resistance through lncRNA expression and Erk phosphorylation; furthermore, there was evidence for a phenyl sulfate correlation with insulin resistance (measured via the urinary C-peptide/creatinine ratio) in two CKD patient cohorts [45].

In terms of brain health, gut-derived uremic toxins incur neuroinflammation, blood–brain barrier dysfunction and cerebral microvascular disease in CKD [46,47,48,49]. Uremic serum exposure of brain endothelial cells in vitro downregulates key tight junction proteins, such as claudin-5 and occludin, and induces cytoskeletal rearrangements, culminating in the breakdown of the endothelial barrier [46,47]. Indoxyl sulfate in particular has been shown to disrupt the blood–brain barrier and to cause neurodegeneration in CKD rodent models [50,51] and is associated with cognitive impairment in CKD patients [52]. Cerebral microvascular disease is the major precursor to stroke and cognitive decline in CKD [53,54] and includes microbleeds ≤ 10 mm detected on magnetic resonance imaging (MRI). The prevalence of microbleeds increases from 14% in CKD stage 3 to 34% in CKD stage 5 [55], to ~50% in ESKD patients [56,57,58,59]. In aged CKD animals, gut dysbiosis with the associated elevated serum levels of gut-derived toxins is particularly pronounced, whereby indoxyl sulfate and TMAO correlate with a higher burden of brain microbleeds [60].

## 3. Influence of Diet on the Microbiota

### 3.1. Protein Intake

There is a lack of consensus regarding the formal definition of a high-protein diet, with a reported range between 1.2 and 2.0 g/kg/day [61]. High dietary protein intake can incur proteinuria and an initial rise in the glomerular filtration rate (GFR) via hyperfiltration, with loss of kidney function over time due to pro-inflammatory and fibrosis pathways [61,62]. Further, this protein intake leads to an amino acid load which can promote a proteolytic rather than saccharolytic gut microbiome, enhancing the generation of gut-derived uremic toxins. As noted above, the major bacterial-derived toxins indoxyl sulfate, p-cresyl sulfate and TMAO are derived from amino acids and quarternary amines present in dairy, eggs, seafood, meat and soy products. Animal foods, in contrast to plant-source proteins, are perceived to offer better digestibility and bioavailability of proteins and amino acids (higher protein-to-energy ratios) [63]. In particular, red meat has been demonstrated to modulate gut microbiota-dependent generation of TMAO, mediated by the γBB utilization (gbu) microbial gene cluster critical to carnitine metabolism [64]. However, other investigations have shown conflicting findings about increased vs decreased colonic inflammation downstream of the microbiota changes induced by red meat intake [65]. Yet other studies have examined choline intake from eggs and reported no increase in blood TMAO levels [65]. It is important to note that these investigations involved cohorts of healthy individuals or patients with metabolic syndrome or inflammatory bowel disease; further research is needed to elucidate the impact of dietary protein on the gut microbiota and circulating gut-derived toxins in the CKD population.

### 3.2. Sugars and Sweeteners

The amount and type of dietary sugars and sweeteners can alter the gut microbiota. Excess sugar (glucose, fructose) intake has been associated with an increase in the gut flora *Proteobacteria*, with a concurrent reduction in *Bacteroidetes*, a breakdown of the intestinal epithelial barrier and endotoxemia [66,67]. In particular, fructose is not absorbed in the small intestine and the passage of a fructose osmotic load to the colon provides a substrate for bacterial fermentation, thereby altering the microbial population [68]. A longitudinal study in the UK Biobank database, where participants were followed for a median of 10.5 years, reported an increased risk of incident CKD with a higher intake of sugars, that was in part linked to gut microbial abundance [69]. In terms of non-nutritive sweeteners such as aspartame, sucralose and saccharin, these food additives have been shown to alter beta-diversity and modify the host metabolic phenotype, including increasing the risk of insulin resistance [70,71,72,73]. In vitro co-culture studies demonstrated that sweeteners have a varying impact in promoting biofilm formation by *Escherichia coli* and *Enterococcus faecalis*, incurring injury to the host epithelium [74]. Of note, a clinical trial with 17 health individuals reported that daily consumption of aspartame or sucralose did not alter the gut microbiome nor the amounts of fecal SCFAs [75], and dedicated trials are needed to fully elucidate the impact of non-nutritive sweeteners on the gut microbiome in CKD patients.

### 3.3. Dietary Fats

The quantity of dietary fats and their saturation (presence of double bonds between carbon molecules) modulate the gut microbiota, which metabolize these fats into various fatty acids [10]. Polyunsaturated fatty acids (PUFAs), found in sunflower oil, fatty fish, nuts and seeds are deemed “essential fatty acids” that need to be obtained from the diet, as they are not synthesized by the human body. Omega-3 PUFAs can beneficially restore the gut *Firmicutes*/*Bacteroidetes* ratio and increase Lachnospiraceae which produce the SCFA butyrate [10], and a low intake of saturated fatty acids is linked with a higher microbial diversity in humans [76]. Conversely, the typical Western diet is high in saturated fats and omega-6 PUFAs (sunflower and corn oil, processed foods, eggs, animal fats) and is associated with an impaired gut endothelial barrier and endotoxemia [77], concurrent with an increased risk of metabolic syndrome and cardiovascular disease.

Moderate intake of monounsaturated fatty acids (MUFAs, palmitoleic, oleic and eicosenoic fatty acids, found in sesame, pumpkin seeds, extra virgin olive oil and peanuts) and medium-chain fatty acids (MCFAs, found in coconut oil and human milk) has been associated with positive effects pertaining to increased gut microbiota diversity and improved host metabolic functions [10,78]. However, a high intake of MCFA-rich coconut oil increases the *Firmicutes* to *Bacteroidetes* ratio as well as increases gut Clostridium and Staphylococcus [10], and in a rat model, it was observed to increase hepatic fatty acid deposition and adipose tissue inflammation [79]. Animal products such as lard, bacon, fatty red meat and dairy products are rich in saturated fatty acids, which can increase the relative proportion of gut Faecalibacterium and *Firmicutes*, disrupting the *Bacteroidetes*: *Firmicutes* balance [80]. While a high-fat diet is associated with inflammation and renal proximal tubular injury [81], the impact of dietary fats on the gut microbiome in CKD has yet to be studied.

### 3.4. Fiber Intake

Dietary fibers include non-starch polysaccharides, oligosaccharides, and resistant starch. Undigested carbohydrates that are derived from these dietary fibers go through anaerobic fermentation by bacteria (primarily *Bacteroidetes* and *Firmicutes* phyla) to produce SCFAs which, as noted above, are energy sources for colonic epithelial cells and have receptor-mediated signaling on tight junction proteins, which regulate the integrity of the gut epithelial barrier and limit the systemic translocation of gut-derived uremic toxins [4,82].

The CKD diet tends to restrict potassium, which leads to a lower intake of fiber-rich plant-based foods, including vegetables, fruits, legumes, nuts, and whole grains. This long-standing dietary guidance is being reconsidered, as recent medical literature has found that dietary potassium has no or a weak association with serum potassium levels and hyperkalemia in CKD patients [83,84]. A meta-analysis of 19,843 participants indicates that increasing dietary fiber consumption reduces all-cause mortality, cardiovascular mortality, and cardiovascular disease in CKD patients [85]. The Kidney Disease: Improving Global Outcomes (KDIGO) CKD practice guidelines state that the net bioavailable potassium from plant-based foods is lower than from highly processed foods, meats, dairy products, juices, and salt substitutes made with potassium chloride [86]. A fiber-rich diet can also reduce populations of uremic toxin-producing organisms [87]. CKD mouse studies have shown that supplemental feeding with fermentable dietary fibers may prevent or manage CKD by restoring colonic barrier integrity and microflora composition [88].

### 3.5. Polyphenols

Polyphenols are compounds found in plant-based foods, characterized chemically by multiple phenol units. These compounds include flavonoids, tannins, phenolic acids, and their derivatives. Once polyphenols from the diet reach the gut, the microbiota metabolizes the polyphenols into bioactive compounds that cross the intestinal barrier and mitigates oxidative stress, inflammation, and fibrosis, including in the kidney. Polyphenols also directly affect the composition of the gut microbiota, increasing beneficial bacteria and inhibiting the proliferation of pathogenic bacteria [89].

Oxidative stress is commonly observed in CKD patients due to the overproduction of reactive oxygen species and the impairment of defense mechanisms. Polyphenols exhibit antioxidant and anti-inflammatory properties and theoretically can prevent or inhibit the progression of CKD, with promising findings from preclinical studies demonstrating that several polyphenols (punicalagin, resveratrol, emodin, magnesium lithospermate B, epigallocatechin gallate, chlorogenic acid, fisetin, luteolin, curcumin) foster a beneficial gut microbiome [90,91].

There is limited clinical data about polyphenols in CKD, with most papers having small sample sizes, short follow-up, and variability in polyphenol type and dosing. Studies on curcumin in dialysis patients found a decrease in p-cresyl sulfate and markers of inflammation [90]. However, Alvarenga et al. reported that supplementation with trans-resveratrol did not reduce the plasma levels of inositol sulfate, p-cresyl sulfate, and indole-3-acetic acid in pre-dialysis CKD patients [92]. A small study with 33 patients found that grape seed extract improved oxidative stress, GFR and proteinuria [93]. A meta-analysis of polyphenol interventions in diabetic nephropathy has shown statistically significant, but clinically moderate, improvements in HbA1c, proteinuria, and GFR [94]. Further studies are warranted to elucidate the health effects of dietary polyphenols and their potential for drug development, where challenges include cytochrome P450-mediated drug interactions, and uncertainties surrounding appropriate dosing and long-term safety [90].

### 3.6. Nutritional Supplements

Nutritional supplements commonly taken by CKD patients that affect the gut microbiome include iron supplements, vitamin supplementation, and ketoanalogues of amino acids. Iron supplementation is commonly given to CKD patients for iron deficiency anemia, though its efficacy is limited as chronic inflammation in CKD elevates hepcidin levels, which blocks intestinal iron absorption and iron release from storage sites [95]. Studies examining the effects of iron supplementation on the gut microbiota and the epithelial barrier have shown mixed findings [96]. Ferric citrate, an oral iron supplement that also serves as a phosphate binder, was reported in a rat CKD model to increase gut microbial diversity and the abundance of beneficial taxa, without increasing the levels of uremic toxins [24]. In contrast, Xia et al. found that ferric citrate induced injury of the colonic epithelial barrier in healthy mice [97]. One prospective study in hemodialysis patients reported that oral iron decreased alpha-diversity in the gut and SCFA-producing bacteria; however, it also increased beneficial *Lactobacillus* species compared to intravenous iron [98]. Overall, the clinical significance of microbiome alterations with iron supplementation remains uncertain, and iron supplementation should be used judiciously.

The KDOQI guidelines recommend the supplementation of water-soluble vitamins in dialysis patients with inadequate dietary intake [99]. A review by Wang et al. recommends individualized vitamin D supplementation based on serum vitamin D and parathyroid hormone levels; however, vitamin A and E supplementation is not supported due to potential toxicity, and vitamin K supplementation lacks robust evidence for clinical benefit [100]. Despite these supplementation recommendations, little is known about how vitamin supplementation may influence gut microbiota composition or function in CKD patients. Evidence from non-CKD populations suggests that vitamin D supplementation can enhance gut microbiome diversity [101], underscoring the need for further investigation in the CKD population.

Ketoanalogues (KAs) of amino acids are typically used to supplement protein-restricted diets in CKD patients. These compounds are designed to be transaminated in vivo to their corresponding amino acids, which uses endogenous nitrogen sources and avoids producing nitrogenous waste [102]. The KDIGO guidelines note that KAs may confer benefits that include slowing GFR decline and reducing metabolic acidosis; however, the evidence is limited due to issues with medication adherence from a high pill burden and high cost [86]. Relating to gut microbiome effects, CKD rats given KAs supplementation were found to have an improved intestinal barrier concurrent with increased beneficial gut bacteria (Methanobrevibacter, Akkermansia, Blautia and Anaerositipes) and reduced numbers of potentially harmful bacteria (Anaerovorax and Coprococcus_3) [102]. Clinical studies found that very-low-protein diets or a Mediterranean diet supplemented with KAs significantly reduced p-cresyl sulfate and indoxyl sulfate [103,104].

### 3.7. Dietary Salt

Excessive sodium intake is associated with elevated blood pressure and cardiovascular events, leading the World Health Organization (WHO) to recommend limiting sodium intake to <2 g daily to mitigate cardiovascular risk [105]. High salt also exacerbates CKD progression via inducing proteinuria and glomerular hyperfiltration [105]. Animal studies have demonstrated that a high-salt diet can modify the gut microbiome in ways that could interact with CKD progression. Mice fed a high-salt diet showed a decrease in gut Lactobacillus, *Bacteroidetes* and *Proteobacteria*, while *Firmicutes* was increased [106,107]. In 5/6-nephrectomized CKD rats, a high-salt diet was associated with reduced claudin-4 and occludin tight junction proteins in the colon (i.e., a “leaky gut”), concurrent with higher plasma concentrations of the uremic toxins indoxyl sulfate and p-cresyl sulfate [108]. Finally, evidence from a mouse CKD model [109] as well as from a randomized controlled trial with non-CKD hypertensive patients in the United Kingdom [110], supports a link between a high-salt diet and decreased levels of beneficial SCFAs. Collectively, the data suggests that salt restriction can have benefits beyond ameliorating hypertension, pertaining to decreased gut dysbiosis and improved SCFAs production.

## 4. Gut-Directed Interventions

### 4.1. Dietary Styles

We recommend the review by Rinninella et al. for a succinct overview of various dietary styles (Mediterranean, Western, ketogenic, gluten-free, vegan/vegetarian) and their impact on the gut microbiota in the general population [10]. Conventional CKD diet counseling emphasizes the restriction of sodium, potassium and phosphate; however, as noted above, this potassium and phosphate restriction leads to a decreased intake of vegetables, dairy and yogurt, which compromises food choices and alters the gut microbiota [4,14]. The Mediterranean diet is a well-characterized anti-inflammatory diet encompassing plant-based foods, healthy fats (notably olive oil) and fish, with limited red meat and processed foods, and it has been proposed as a beneficial medical nutrition therapy in CKD. While its gut microbiota effects have not been studied specifically in the CKD population, a meta-analysis of 10 studies in non-dialysis CKD cohorts reported benefits with the Mediterranean diet in reducing C-reactive protein, improving body composition and slowing the kidney function decline [111]. There were no significant changes in blood pressure, lipid profiles, or serum potassium or phosphate levels [111]. It has been proposed that the higher fiber intake with the Mediterranean diet improves gut microbial diversity (saccharolytic fermentation rather than proteolytic activity) and increases the production of anti-inflammatory SCFAs, while decreasing uremic toxin generation [111,112].

Conversely, there are concerns about the ketogenic diet (a popular weight-loss strategy) which is characterized by a low-carbohydrate and high-fat intake, generally with a high-protein content. The increased dietary acid and protein intake may exacerbate metabolic acidosis, hyperkalemia, protein catabolism and kidney function decline [113]. In a rat model, the ketogenic diet altered the microbiome and decreased the gut microbial production of SCFAs, concurrent with hepatic lipid accumulation and glucose intolerance [114]. In contrast, studies in obese individuals noted that the ketogenic diet improved lipid and glucose tolerance indices, though with a slight worsening of kidney function; there was an increase in the *Bacteroidetes*-to-*Firmicutes* ratio, though gut microbial changes were not sustained in the weight maintenance phase [115,116]. Autosomal dominant polycystic kidney disease is one specific CKD etiology where ketogenic diets have shown promising effects in terms of preserving kidney function via slowing cyst growth (as the cyst-lining cells are unable to metabolize ketone bodies as fuel) [113,117,118]. More studies are needed to elucidate the benefits vs risks of the ketogenic diet in CKD, though overall it appears to be a feasible intervention for obesity with the judicious monitoring of protein intake and the minimization of commercially processed products [113].

**Figure 1:**
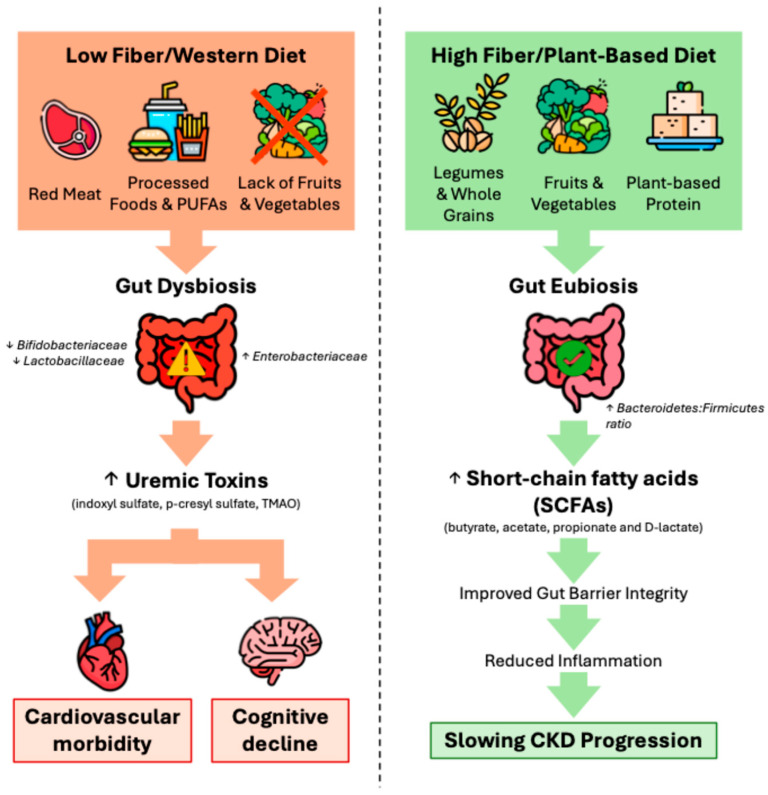
Effect of dietary patterns on the gut microbiome in chronic kidney disease (CKD). The Western diet—high in animal protein, processed foods and polyunsaturated fatty acids (PUFAs), while being low in fiber due to lack of fruits/vegetables—promotes gut dysbiosis. This increases levels of gut-derived uremic toxins, which translocate into the bloodstream and lead to cardiovascular morbidity and cognitive decline downstream. In contrast, a plant-based fiber-rich diet fosters eubiosis and higher levels of short-chain fatty acids (SCFAs), which improve gut barrier integrity, reduces systemic inflammation, and can slow CKD progression.

Overall, plant-based dietary approaches appear promising to slow CKD progression, with salutary effects on renal hemodynamics, the nitrogen metabolic load, uremic toxins, acid–base status and inflammation and oxidative stress (Figure 1) [119,120]. While the microbiome impact of strict vegan/vegetarian diets has not been rigorously studied in CKD patients, the increased fiber intake and the shift to saccharolytic fermentation, with a lower phosphate bioavailability and a dietary acid load, collectively contribute to the reduced generation of gut-derived toxins [119,121]. In a randomized crossover trial involving 25 Australian adults with CKD stage 3–4, a high-diversity plant-based diet (≥30 compared to ≤15 unique plant foods weekly) shifted the gut microbiome toward the increased production of beneficial SCFAs, concurrent with a reduced acid load; the gut-derived uremic toxins indoxyl sulfate and p-cresyl sulfate were decreased in those with more advanced CKD and higher baseline toxin levels [122]. Thus, a plant-dominant diet especially in advanced CKD appears to offer microbial diversity and host metabolic benefits.

The Plant-Dominant Low-Protein Diet (PLADO) [119] represents a pragmatic, patient-centered evolution of traditional low-protein diets, emphasizing predominantly plant-based protein sources while allowing modest amounts of animal protein to enhance adherence and nutritional adequacy. PLADO is typically prescribed at approximately 0.6–0.8 g/kg/day of protein, with ≥50–70% derived from plant sources. Early observational and interventional data suggest favorable effects on metabolic acidosis, phosphorus control, blood pressure, and potentially CKD progression [123,124]. The Plant-Focused Nutrition in Diabetic Kidney Disease (PLAFOND) is a sub-type of the PLADO diet that emphasizes > 50% plant-based protein sources, high dietary fiber, low glycemic index, and 25–35 Cal/kg/day energy [125].

### 4.2. Oral Adsorbents (Including AST-120)

Oral adsorbents represent a gut-directed therapeutic strategy aimed at reducing systemic exposure to uremic toxins by interrupting their intestinal generation and absorption [126,127]. The oral adsorbent that has been most extensively studied is AST-120 (Kremezin), a spherical carbon adsorbent that binds low-molecular-weight organic compounds and uremic toxin precursors in the gastrointestinal tract, thereby reducing the circulating levels of protein-bound toxins. Clinical trials of AST-120 have yielded mixed results, with some large randomized studies failing to meet primary endpoints related to hard renal outcomes. However, subgroup and observational analyses suggest a potential benefit in selected populations, particularly when therapy is initiated earlier in CKD, adherence is high, and the background dietary protein intake is controlled [128]. A post hoc analysis of the K-STAR study (Kremezin study against renal disease progression in Korea) reported a lowering of serum indoxyl sulfate in conjunction with a reduction in CKD progression and cardiovascular events [129].

Importantly, AST-120s mechanistic rationale aligns closely with the contemporary understanding of the kidney–gut axis, suggesting that patient selection and combination strategies may be critical determinants of efficacy. One such strategy involves combining oral adsorbents with low-protein diets, which offers a dual mechanism: reduced toxin generation and enhanced intestinal binding of remaining precursors [130]. In summary, while oral adsorbents such as AST-120 are unlikely to represent stand-alone solutions, their role as part of a broader, diet-centered, gut-focused therapeutic framework warrants renewed attention.

### 4.3. Prebiotics, Probiotics and Synbiotics

Dietary supplementation with prebiotics, probiotics, and synbiotics is increasingly recognized as a promising strategy to modulate the gut microbiota in patients with CKD. In a recent Cochrane review of 45 randomized controlled trials (n = 2266 participants) examining the benefits and harms of prebiotics, probiotics, and synbiotics for patients across all stages of CKD [131], researchers found very low-certainty evidence with no clear benefits of these supplements on survival, kidney function, or other major clinical outcomes, although some small studies suggested reductions in inflammatory markers (e.g., C-reactive protein and interleukin-6) and uremic toxins (e.g., indoxyl sulphate and p-cresyl sulfate). Adverse events were generally rare and mild [131]. A more recent systematic review and network meta-analysis of randomized trials from 2019 to 2023 involving 331 patients with CKD stages 3–5 found evidence supporting the potential benefits of the gut microbiome modulation with prebiotics and probiotics in reducing indoxyl sulfate and p-cresyl sulfate [132]. Specifically, for example, prebiotics achieved a surface under the cumulative ranking curve (SUCRA) value of 72.6% for reducing p-cresyl sulfate, compared with 66.2% for probiotics (SUCRA values range from 0% to 100%, with higher scores indicating greater relative effectiveness among interventions). For free p-cresyl sulfate, the SUCRA values were 78.9% for prebiotics and 63.8% for probiotics. Probiotics were particularly effective for both free and total indoxyl sulfate, with SUCRA values of 83.1% and 88.5%, respectively. Prebiotics also demonstrated a SUCRA value of 74.6% for lowering urea levels [132].

Regarding the biotic intake for the gut microbiota modulation, evidence suggests that prebiotic doses above 5 g per day can improve microbial diversity, while higher doses (15–20 g per day) may be required to lower the concentrations of uremic toxins [133]. In contrast, the wide variation in probiotic strains and dosages across studies introduces an uncertainty about the optimal formulations and dosing for patients with CKD. At present, there is insufficient evidence to confirm the efficacy of these supplements in improving hard clinical outcomes (e.g., cardiovascular events and mortality) or to determine whether one type of supplement is superior to another. Well-designed, adequately powered clinical trials are therefore needed to determine the most effective formulations, dosages, and strains to address gut dysbiosis and improve the clinical outcomes in this population.

### 4.4. Fecal Microbiota Transplantation

Fecal microbiota transplantation (FMT) involves transferring gut microbiota from healthy donors to patients with gut dysbiosis, most commonly through orally administered, encapsulated preparations [134]. While FMT has long been established as a second-line therapy for recurrent *Clostridium difficile* infection, its use has more recently expanded to various conditions, including CKD, with the aim of restoring gut homeostasis and reducing the disease risk [134]. In an adenine-induced CKD mouse model, FMT from healthy mice led to higher gut microbiota α-diversity, lower p-cresyl sulfate levels, and improved glucose tolerance compared with untreated CKD mice [135]. Other animal studies have also reported the protective effects of FMT on glomerular and tubulointerstitial injury in diabetic kidney disease [136,137]. Although clinical evidence is still limited, two earlier case reports of patients with IgA nephropathy and membranous nephropathy found that FMT improved kidney function and reduced albuminuria without serious adverse effects [138,139]. In one case of IgA nephropathy [138], FMT was administered 40 times (200 mL daily, 5 days per week) following the protocol described by Paramsothy et al. [140], followed by an additional 57 applications (200 mL daily, 10–15 days per month) over the subsequent five months using stool from two healthy donors. After the treatment, the patient achieved a partial clinical remission, including a 37% reduction in 24 h urinary protein from baseline [138].

More recently, researchers conducted a single-center, double-blind, randomized, placebo-controlled clinical trial (NCT04361097) to evaluate the safety and efficacy of FMT (vs. placebo) in a total of 28 patients with CKD stages 2–4 [141]. They demonstrated that, regardless of the CKD stage, FMT was associated with a slower CKD progression, fewer patients who experienced a decline in eGFR < −1 mL/min/1.73 m^2^ compared to placebo (13.3% vs. 53.8%), and only mild to moderate gastrointestinal adverse events (abdominal distention, diarrhea, constipation, increased frequency of bowel movements, and flatulence). FMT also altered the gut microbiota composition, decreasing *Firmicutes* and *Actinobacteria* while increasing *Bacteroidetes*, *Proteobacteria*, and *Roseburia* species [141]. While these results suggest potential renoprotective benefits of FMT in patients with CKD, the efficacy, safety, and overall risk–benefit profiles of FMT in these patients need to be confirmed in future larger clinical trials.

### 4.5. Exercise

Growing evidence suggests that exercise can increase gut microbial diversity and promote the growth of symbiotic or commensal gut microbes in experimental models and in the non-CKD populations [142,143,144]. In a cross-sectional study of 41 healthy young adults, higher physical fitness levels were associated with greater gut microbial diversity, independent of diet [145]. Fit individuals also had a gut microbiota enriched with butyrate-producing taxa, including *Clostridiales*, *Erysipelotrichaceae*, *Lachnospiraceae*, and *Roseburia*, resulting in increased fecal butyrate, a SCFA linked to improved gut health; this suggests that exercise could serve as a nonpharmacological strategy to enhance the gut microbiota and mitigate dysbiosis-related conditions [145]. Supporting these findings, a recent meta-analysis of 25 randomized controlled or crossover studies involving 1044 adults found that exercise interventions, compared with controls (e.g., rest, health education, or usual care), significantly increased gut microbial α diversity and altered microbiota compositions, including an increase in *Firmicutes* and a decrease in *Bacteroidetes*, indicating a shift in the *Firmicutes*/*Bacteroidetes* ratio [144].

Importantly, despite the potential benefits of exercise on chronic inflammatory conditions through the modulation of the gut microbiota [146], evidence on its effects in the CKD population remains limited, to our knowledge, with only one published study to date. In a randomized controlled trial, three months of aerobic exercise (three times per week) and six months of resistance training (three times per week) were evaluated for their effects on the plasma levels of uremic toxins (i.e., indoxyl sulfate, p-cresyl sulfate, and indole-3-acetic acid) in 20 and 26 hemodialysis patients, respectively [147]. The study found no significant reduction in these uremic toxin levels [147]. In this context, a recent clinical trial (ClinicalTrials.gov identifier: NCT03689569) investigating the combined effects of exercise training and the consumption of a prebiotic supplement (resistant starch) on key inflammatory markers and gut microbiota composition may offer novel insights into this area.

## 5. Conclusions

**Figure 2:**
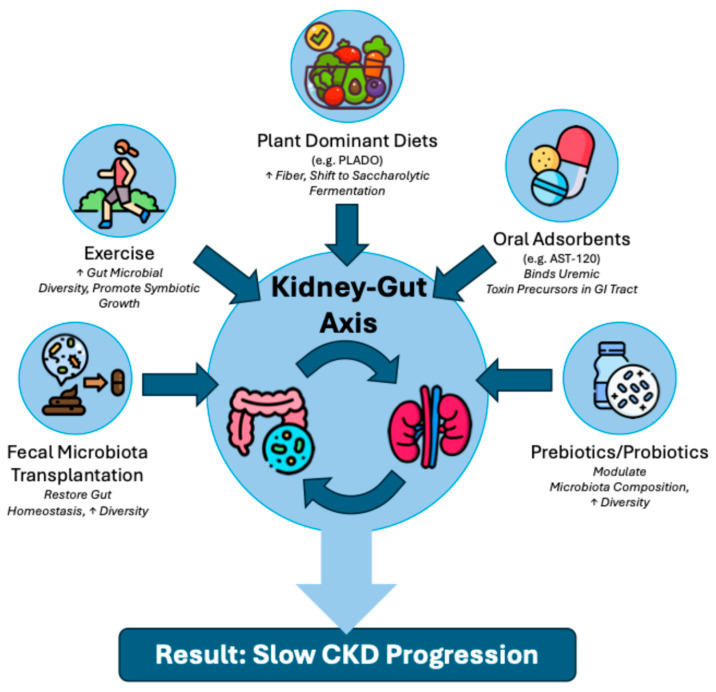
Gut-targeted therapies to modulate the kidney–gut axis and slow CKD. An overview of emerging microbiota-modulating interventions that aim to delay CKD complications by restoring intestinal homeostasis. These therapeutic strategies include: plant-dominant diets (e.g., PLADO) to promote beneficial saccharolytic fermentation; oral adsorbents (e.g., AST-120) to bind low-molecular-weight toxin precursors in the gastrointestinal tract; prebiotics and probiotics to enhance gut microbial diversity; fecal microbiota transplantation to restore a healthy commensal flora; and physical exercise to independently enrich symbiotic microbial growth and diversity.

Gut dysbiosis is a well-recognized component of the bidirectional kidney–gut axis in CKD. Retained waste products alter microbial populations, which in turn generate gut-derived toxins that translocate into the bloodstream and induce systemic pathology. While there is an abundance of data from the general population pertaining to the modulation of the gut microbiome by dietary protein, fiber, sugars and fats, more work is needed to clarify diet-induced microbiota and systemic changes in the CKD population. However, it is likely that diet–microbiome interactions have meaningful implications on CKD outcomes, alongside clinical factors (diabetes, hypertension, proteinuria, smoking, etc.) and the psychosocial determinants of health. Gut-targeted therapies show promise in reducing toxin production and slowing CKD progression (Figure 2)—in particular, a plant-based diet combined with an oral adsorbent poses an appealing combination therapy with a low risk of adverse effects. Future research should explore the impact of nutrition on microorganisms beyond bacteria, such as fungi, archaea, and viruses, to enhance our understanding of the complex diet–gut–kidney axis and facilitate more personalized and effective treatments.

## Figures and Tables

**Table 1:** Gut microbiota in health, pre-dialysis chronic kidney disease (CKD), end-stage kidney disease (hemodialysis and peritoneal dialysis), and kidney transplantation [6,18]. * Decrease in *Lactobacillaceae* and *Prevotellaceae* leads to decreased production of short-chain fatty acids.

**Healthy Gut Microbiome**	90% *Firmicutes* and *Bacteroidetes* (predominantly *Firmicutes*) 10% *Proteobacteria*, Actinobacteria, Euryarchaeota, Verrucomicrobia	

**CKD and Kidney Replacement Strategies**	**Increase/Expansion**	**Decrease/Reduction**
Pre-dialysis CKD	*Enterobacteriae* *Enterococci* *Lachnospiraceae* *Ruminococcaceae*	*Lactobacillaceae* * Prevotellaceae * Bacteroidaceae *Bifidobacterium* species
Hemodialysis	*Firmicutes* (mainly Clostridium, Enterococcus) *Proteobacteria* (mainly Gamma*Proteobacteria*) Actinobacteria	*Lactobacillaceae* * Prevotellaceae *
Peritoneal dialysis	*Proteobacteria* (Pseudomonas aeruginosa)	*Actinobacteria* *Firmicutes* *Lactobacillaceae* * Bifidobacterium species
Kidney transplantation	*Proteobacteria*	*Actinobacteria*

## Data Availability

No new data were created or analyzed in this study.

## Abbreviations

The following abbreviations are used in this manuscript:

CKD	Chronic kidney disease
SCFAs	Short-chain fatty acids
ESKD	End-stage kidney disease
TMAO	Trimethylamine-N-oxide
MRI	Magnetic resonance imaging
eGFR	Estimated glomerular filtration rate
MCFAs	Medium chain fatty acids
KDIGO	Kidney Disease: Improving Global Outcomes
KAs	Ketoanalogues
PLADO	Plant-Dominant Low-Protein Diet
PLAFOND	Plant-Focused Nutrition in Diabetic Kidney Disease
SUCRA	surface under the cumulative ranking curve
FMT	Fecal microbiota transplantation

## References

[1] Mark P.B., Stafford L.K., Grams M.E., Aalruz H., Abd ElHafeez S., Abdelgalil A.A., Abdulkader R.S., Abeywickrama H.M., Abiodun O.O., Abramov D. (2025). Global, regional, and national burden of chronic kidney disease in adults, 1990–2023, and its attributable risk factors: A systematic analysis for the Global Burden of Disease Study 2023. Lancet.

[2] Bikbov B., Purcell C.A., Levey A.S., Smith M., Abdoli A., Abebe M., Adebayo O.M., Afarideh M., Agarwal S.K., Agudelo-Botero M. (2020). Global, regional, and national burden of chronic kidney disease, 1990–2017: A systematic analysis for the Global Burden of Disease Study 2017. Lancet.

[3] Matsushita K., Ballew S.H., Wang A.Y., Kalyesubula R., Schaeffner E., Agarwal R. (2022). Epidemiology and risk of cardiovascular disease in populations with chronic kidney disease. Nat. Rev. Nephrol..

[4] Lau W.L., Savoj J., Nakata M.B., Vaziri N.D. (2018). Altered microbiome in chronic kidney disease: Systemic effects of gut-derived uremic toxins. Clin. Sci..

[5] Lau W.L., Vaziri N.D. (2017). The Leaky Gut and Altered Microbiome in Chronic Kidney Disease. J. Ren. Nutr..

[6] Tourountzis T., Lioulios G., Fylaktou A., Moysidou E., Papagianni A., Stangou M. (2022). Microbiome in Chronic Kidney Disease. Life.

[7] Savage D.C. (1977). Microbial ecology of the gastrointestinal tract. Annu. Rev. Microbiol..

[8] Lozupone C.A., Stombaugh J.I., Gordon J.I., Jansson J.K., Knight R. (2012). Diversity, stability and resilience of the human gut microbiota. Nature.

[9] Ianiro G., Iorio A., Porcari S., Masucci L., Sanguinetti M., Perno C.F., Gasbarrini A., Putignani L., Cammarota G. (2022). How the gut parasitome affects human health. Ther. Adv. Gastroenterol..

[10] Rinninella E., Tohumcu E., Raoul P., Fiorani M., Cintoni M., Mele M.C., Cammarota G., Gasbarrini A., Ianiro G. (2023). The role of diet in shaping human gut microbiota. Best Pract. Res. Clin. Gastroenterol..

[11] Nyangahu D.D., Jaspan H.B. (2019). Influence of maternal microbiota during pregnancy on infant immunity. Clin. Exp. Immunol..

[12] Arumugam M., Raes J., Pelletier E., Le Paslier D., Yamada T., Mende D.R., Fernandes G.R., Tap J., Bruls T., Batto J.M. (2011). Enterotypes of the human gut microbiome. Nature.

[13] Lau W.L., Vaziri N.D. (2017). Urea, a true uremic toxin: The empire strikes back. Clin. Sci..

[14] Su G., Qin X., Yang C., Sabatino A., Kelly J.T., Avesani C.M., Carrero J.J. (2022). Fiber intake and health in people with chronic kidney disease. Clin. Kidney J..

[15] Anders H.J., Andersen K., Stecher B. (2013). The intestinal microbiota, a leaky gut, and abnormal immunity in kidney disease. Kidney Int..

[16] Wong J., Piceno Y.M., Desantis T.Z., Pahl M., Andersen G.L., Vaziri N.D. (2014). Expansion of urease- and uricase-containing, indole- and p-cresol-forming and contraction of short-chain fatty acid-producing intestinal microbiota in ESRD. Am. J. Nephrol..

[17] Vaziri N.D., Wong J., Pahl M., Piceno Y.M., Yuan J., Desantis T.Z., Ni Z., Nguyen T.H., Andersen G.L. (2013). Chronic kidney disease alters intestinal microbial flora. Kidney Int..

[18] Madhogaria B., Bhowmik P., Kundu A. (2022). Correlation between human gut microbiome and diseases. Infect. Med..

[19] Rumanli Z., Vural I.M., Alp Avci G. (2025). Chronic kidney disease, uremic toxins and microbiota. Microbiota Host.

[20] Jackson M.A., Goodrich J.K., Maxan M.E., Freedberg D.E., Abrams J.A., Poole A.C., Sutter J.L., Welter D., Ley R.E., Bell J.T. (2016). Proton pump inhibitors alter the composition of the gut microbiota. Gut.

[21] Rahbar Saadat Y., Niknafs B., Hosseiniyan Khatibi S.M., Ardalan M., Majdi H., Bahmanpoor Z., Abediazar S., Zununi Vahed S. (2020). Gut microbiota; an overlooked effect of phosphate binders. Eur. J. Pharmacol..

[22] Mao Z.H., Liu Y., Pan S., Zhang Q., Qiao Y., Zhang X., Li D., Chen J., Liu D., Feng Q. (2025). The Gut-Kidney Dialogue: Unraveling the Microbial Symphony in Renal Fibrosis. Faseb J..

[23] Kortman G.A.M., Reijnders D., Swinkels D.W. (2017). Oral iron supplementation: Potential implications for the gut microbiome and metabolome in patients with CKD. Hemodial. Int..

[24] Lau W.L., Vaziri N.D., Nunes A.C.F., Comeau A.M., Langille M.G.I., England W., Khazaeli M., Suematsu Y., Phan J., Whiteson K. (2018). The Phosphate Binder Ferric Citrate Alters the Gut Microbiome in Rats with Chronic Kidney Disease. J. Pharmacol. Exp. Ther..

[25] Tang Y., Li Y., Yang X., Lu T., Wang X., Li Z., Liu J., Wang J. (2026). Intestinal metabolite TMAO promotes CKD progression by stimulating macrophage M2 polarization through histone H4 lysine 12 lactylation. Cell Death Differ..

[26] Miyazaki T., Ise M., Hirata M., Endo K., Ito Y., Seo H., Niwa T. (1997). Indoxyl sulfate stimulates renal synthesis of transforming growth factor-beta 1 and progression of renal failure. Kidney Int. Suppl..

[27] Watanabe H., Miyamoto Y., Honda D., Tanaka H., Wu Q., Endo M., Noguchi T., Kadowaki D., Ishima Y., Kotani S. (2013). p-Cresyl sulfate causes renal tubular cell damage by inducing oxidative stress by activation of NADPH oxidase. Kidney Int..

[28] Ichii O., Otsuka-Kanazawa S., Nakamura T., Ueno M., Kon Y., Chen W., Rosenberg A.Z., Kopp J.B. (2014). Podocyte injury caused by indoxyl sulfate, a uremic toxin and aryl-hydrocarbon receptor ligand. PLoS ONE.

[29] Jankowski J., Floege J., Fliser D., Böhm M., Marx N. (2021). Cardiovascular Disease in Chronic Kidney Disease. Circulation.

[30] Webster A.C., Nagler E.V., Morton R.L., Masson P. (2017). Chronic Kidney Disease. Lancet.

[31] Masai N., Tatebe J., Yoshino G., Morita T. (2010). Indoxyl sulfate stimulates monocyte chemoattractant protein-1 expression in human umbilical vein endothelial cells by inducing oxidative stress through activation of the NADPH oxidase-nuclear factor-κB pathway. Circ. J..

[32] Kharait S., Haddad D.J., Springer M.L. (2011). Nitric oxide counters the inhibitory effects of uremic toxin indoxyl sulfate on endothelial cells by governing ERK MAP kinase and myosin light chain activation. Biochem. Biophys. Res. Commun..

[33] Delgado-Marin M., Sánchez-Esteban S., Cook-Calvete A., Jorquera-Ortega S., Zaragoza C., Saura M. (2024). Indoxyl Sulfate-Induced Valve Endothelial Cell Endothelial-to-Mesenchymal Transition and Calcification in an Integrin-Linked Kinase-Dependent Manner. Cells.

[34] Adelibieke Y., Shimizu H., Muteliefu G., Bolati D., Niwa T. (2012). Indoxyl sulfate induces endothelial cell senescence by increasing reactive oxygen species production and p53 activity. J. Ren. Nutr..

[35] Adijiang A., Higuchi Y., Nishijima F., Shimizu H., Niwa T. (2010). Indoxyl sulfate, a uremic toxin, promotes cell senescence in aorta of hypertensive rats. Biochem. Biophys. Res. Commun..

[36] Yamamoto H., Tsuruoka S., Ioka T., Ando H., Ito C., Akimoto T., Fujimura A., Asano Y., Kusano E. (2006). Indoxyl sulfate stimulates proliferation of rat vascular smooth muscle cells. Kidney Int..

[37] Barreto F.C., Barreto D.V., Liabeuf S., Meert N., Glorieux G., Temmar M., Choukroun G., Vanholder R., Massy Z.A., European Uremic Toxin Work Group (EUTox) (2009). Serum indoxyl sulfate is associated with vascular disease and mortality in chronic kidney disease patients. Clin. J. Am. Soc. Nephrol..

[38] Lin C.J., Liu H.L., Pan C.F., Chuang C.K., Jayakumar T., Wang T.J., Chen H.H., Wu C.J. (2012). Indoxyl sulfate predicts cardiovascular disease and renal function deterioration in advanced chronic kidney disease. Arch. Med. Res..

[39] Cao X.S., Chen J., Zou J.Z., Zhong Y.H., Teng J., Ji J., Chen Z.W., Liu Z.H., Shen B., Nie Y.X. (2015). Association of indoxyl sulfate with heart failure among patients on hemodialysis. Clin. J. Am. Soc. Nephrol..

[40] Koeth R.A., Wang Z., Levison B.S., Buffa J.A., Org E., Sheehy B.T., Britt E.B., Fu X., Wu Y., Li L. (2013). Intestinal microbiota metabolism of l-carnitine, a nutrient in red meat, promotes atherosclerosis. Nat. Med..

[41] Tang W.H., Wang Z., Levison B.S., Koeth R.A., Britt E.B., Fu X., Wu Y., Hazen S.L. (2013). Intestinal microbial metabolism of phosphatidylcholine and cardiovascular risk. N. Engl. J. Med..

[42] Stubbs J.R., House J.A., Ocque A.J., Zhang S., Johnson C., Kimber C., Schmidt K., Gupta A., Wetmore J.B., Nolin T.D. (2016). Serum Trimethylamine-N-Oxide is Elevated in CKD and Correlates with Coronary Atherosclerosis Burden. J. Am. Soc. Nephrol..

[43] Stockler-Pinto M.B., Saldanha J.F., Yi D., Mafra D., Fouque D., Soulage C.O. (2016). The uremic toxin indoxyl sulfate exacerbates reactive oxygen species production and inflammation in 3T3-L1 adipose cells. Free Radic. Res..

[44] Koppe L., Pillon N.J., Vella R.E., Croze M.L., Pelletier C.C., Chambert S., Massy Z., Glorieux G., Vanholder R., Dugenet Y. (2013). p-Cresyl sulfate promotes insulin resistance associated with CKD. J. Am. Soc. Nephrol..

[45] Tongu Y., Kasahara T., Akiyama Y., Suzuki T., Ho H.-J., Matsumoto Y., Kujirai R., Kikuchi K., Nata K., Kanzaki M. (2025). Hypoglycemia and hyperinsulinemia induced by phenolic uremic toxins in CKD and DKD patients. Sci. Rep..

[46] Lau W.L., Nunes A.C.F., Vasilevko V., Floriolli D., Lertpanit L., Savoj J., Bangash M., Yao Z., Shah K., Naqvi S. (2020). Chronic Kidney Disease Increases Cerebral Microbleeds in Mouse and Man. Transl. Stroke Res..

[47] Fang C., Lau W.L., Sun J., Chang R., Vallejo A., Lee D., Liu J., Liu H., Hung Y.H., Zhao Y. (2023). Chronic kidney disease promotes cerebral microhemorrhage formation. J. Neuroinflamm..

[48] Andrews T.D., Day G.S., Irani S.R., Kanekiyo T., Hickson L.J. (2025). Uremic Toxins, CKD, and Cognitive Dysfunction. J. Am. Soc. Nephrol. JASN.

[49] Lee S.H., Ryu J.C., Minasyan M., Lau W.L., Fisher M. (2026). Chronic Kidney Disease and Cerebral Microbleeds: Pathophysiological Insights and Implications for Nephrology. Clin. J. Am. Soc. Nephrol..

[50] Bobot M., Thomas L., Moyon A., Fernandez S., McKay N., Balasse L., Garrigue P., Brige P., Chopinet S., Poitevin S. (2020). Uremic Toxic Blood-Brain Barrier Disruption Mediated by AhR Activation Leads to Cognitive Impairment during Experimental Renal Dysfunction. J. Am. Soc. Nephrol..

[51] Sun C.Y., Li J.R., Wang Y.Y., Lin S.Y., Ou Y.C., Lin C.J., Wang J.D., Liao S.L., Chen C.J. (2021). Indoxyl sulfate caused behavioral abnormality and neurodegeneration in mice with unilateral nephrectomy. Aging.

[52] Yeh Y.C., Huang M.F., Liang S.S., Hwang S.J., Tsai J.C., Liu T.L., Wu P.H., Yang Y.H., Kuo K.C., Kuo M.C. (2016). Indoxyl sulfate, not p-cresyl sulfate, is associated with cognitive impairment in early-stage chronic kidney disease. Neurotoxicology.

[53] Lau W.L., Huisa B.N., Fisher M. (2017). The Cerebrovascular-Chronic Kidney Disease Connection: Perspectives and Mechanisms. Transl. Stroke Res..

[54] Lau W.L., Fisher M. (2022). New insights into cognitive decline in chronic kidney disease. Nat. Rev. Nephrol..

[55] Shima H., Ishimura E., Naganuma T., Yamazaki T., Kobayashi I., Shidara K., Mori K., Takemoto Y., Shoji T., Inaba M. (2010). Cerebral microbleeds in predialysis patients with chronic kidney disease. Nephrol. Dial. Transpl..

[56] Yokoyama S., Hirano H., Uomizu K., Kajiya Y., Tajitsu K., Kusumoto K. (2005). High incidence of microbleeds in hemodialysis patients detected by T2*-weighted gradient-echo magnetic resonance imaging. Neurol. Med. Chir..

[57] Naganuma T., Takemoto Y., Yamasaki T., Shima H., Shoji T., Ishimura E., Nishizawa Y., Morino M., Okamura M., Nakatani T. (2011). Factors associated with silent cerebral microbleeds in hemodialysis patients. Clin. Nephrol..

[58] Chai C., Wang Z., Fan L., Zhang M., Chu Z., Zuo C., Liu L., Mark Haacke E., Guo W., Shen W. (2016). Increased Number and Distribution of Cerebral Microbleeds Is a Risk Factor for Cognitive Dysfunction in Hemodialysis Patients: A Longitudinal Study. Medicine.

[59] Li L., Fisher M., Lau W.L., Moradi H., Cheung A., Thai G., Handwerker J., Kalantar-Zadeh K. (2015). Cerebral microbleeds and cognitive decline in a hemodialysis patient: Case report and review of literature. Hemodial. Int..

[60] Zhao Y., Tran T., Fang C., Paganini-Hill A., Dulkanchainun M., Mai E., Eprem L., Cribbs D., Fisher M., Lau W.L. (2025). Gut dysbiosis and brain microhemorrhages in young vs. aged mice with chronic kidney disease. Sci. Rep..

[61] Ko G.J., Rhee C.M., Kalantar-Zadeh K., Joshi S. (2020). The Effects of High-Protein Diets on Kidney Health and Longevity. J. Am. Soc. Nephrol..

[62] Kalantar-Zadeh K., Fouque D. (2017). Nutritional Management of Chronic Kidney Disease. N. Engl. J. Med..

[63] Górska-Warsewicz H., Laskowski W., Kulykovets O., Kudlińska-Chylak A., Czeczotko M., Rejman K. (2018). Food Products as Sources of Protein and Amino Acids-The Case of Poland. Nutrients.

[64] Buffa J.A., Romano K.A., Copeland M.F., Cody D.B., Zhu W., Galvez R., Fu X., Ward K., Ferrell M., Dai H.J. (2022). The microbial gbu gene cluster links cardiovascular disease risk associated with red meat consumption to microbiota L-carnitine catabolism. Nat. Microbiol..

[65] Lee C., Lee J., Eor J.Y., Kwak M.J., Huh C.S., Kim Y. (2023). Effect of Consumption of Animal Products on the Gut Microbiome Composition and Gut Health. Food Sci. Anim. Resour..

[66] Do M.H., Lee E., Oh M.J., Kim Y., Park H.Y. (2018). High-Glucose or -Fructose Diet Cause Changes of the Gut Microbiota and Metabolic Disorders in Mice without Body Weight Change. Nutrients.

[67] Satokari R. (2020). High Intake of Sugar and the Balance between Pro- and Anti-Inflammatory Gut Bacteria. Nutrients.

[68] Gibson P.R., Newnham E., Barrett J.S., Shepherd S.J., Muir J.G. (2007). Review article: Fructose malabsorption and the bigger picture. Aliment. Pharmacol. Ther..

[69] Zheng G., Zhang Y., Ou F., Chang Q., Ji C., Yang H., Chen L., Xia Y., Zhao Y. (2024). Sugar types, genetic predictors of the gut microbiome, and the risk of chronic kidney disease: A prospective cohort study. Food Funct..

[70] Pepino M.Y. (2015). Metabolic effects of non-nutritive sweeteners. Physiol. Behav..

[71] Markus V., Share O., Shagan M., Halpern B., Bar T., Kramarsky-Winter E., Teralı K., Özer N., Marks R.S., Kushmaro A. (2021). Inhibitory Effects of Artificial Sweeteners on Bacterial Quorum Sensing. Int. J. Mol. Sci..

[72] Kemp J.A., Ribeiro M., Borges N.A., Cardozo L., Fouque D., Mafra D. (2025). Dietary Intake and Gut Microbiome in CKD. Clin. J. Am. Soc. Nephrol..

[73] Gerasimidis K., Bryden K., Chen X., Papachristou E., Verney A., Roig M., Hansen R., Nichols B., Papadopoulou R., Parrett A. (2020). The impact of food additives, artificial sweeteners and domestic hygiene products on the human gut microbiome and its fibre fermentation capacity. Eur. J. Nutr..

[74] Shil A., Chichger H. (2021). Artificial Sweeteners Negatively Regulate Pathogenic Characteristics of Two Model Gut Bacteria, *E. coli* and *E. faecalis*. Int. J. Mol. Sci..

[75] Ahmad S.Y., Friel J., Mackay D. (2020). The Effects of Non-Nutritive Artificial Sweeteners, Aspartame and Sucralose, on the Gut Microbiome in Healthy Adults: Secondary Outcomes of a Randomized Double-Blinded Crossover Clinical Trial. Nutrients.

[76] Schoeler M., Ellero-Simatos S., Birkner T., Mayneris-Perxachs J., Olsson L., Brolin H., Loeber U., Kraft J.D., Polizzi A., Martí-Navas M. (2023). The interplay between dietary fatty acids and gut microbiota influences host metabolism and hepatic steatosis. Nat. Commun..

[77] Malesza I.J., Malesza M., Walkowiak J., Mussin N., Walkowiak D., Aringazina R., Bartkowiak-Wieczorek J., Mądry E. (2021). High-Fat, Western-Style Diet, Systemic Inflammation, and Gut Microbiota: A Narrative Review. Cells.

[78] Roopashree P.G., Shetty S.S., Suchetha Kumari N. (2021). Effect of medium chain fatty acid in human health and disease. J. Funct. Foods.

[79] de Moura e Dias M., Pais Siqueira N., Lopes da Conceição L., Aparecida dos Reis S., Xavier Valente F., Maciel dos Santos Dias M., de Oliveira Barbosa Rosa C., Oliveira de Paula S., da Matta S.L.P., Licursi de Oliveira L. (2018). Consumption of virgin coconut oil in Wistar rats increases saturated fatty acids in the liver and adipose tissue, as well as adipose tissue inflammation. J. Funct. Foods.

[80] Kazura W., Michalczyk K., Stygar D. (2023). The Relationship between the Source of Dietary Animal Fats and Proteins and the Gut Microbiota Condition and Obesity in Humans. Nutrients.

[81] Chen S., Chen J., Li S., Guo F., Li A., Wu H., Chen J., Pan Q., Liao S., Liu H.F. (2021). High-Fat Diet-Induced Renal Proximal Tubular Inflammatory Injury: Emerging Risk Factor of Chronic Kidney Disease. Front. Physiol..

[82] Nogal A., Valdes A.M., Menni C. (2021). The role of short-chain fatty acids in the interplay between gut microbiota and diet in cardio-metabolic health. Gut Microbes.

[83] MacLaughlin H.L., McAuley E., Fry J., Pacheco E., Moran N., Morgan K., McGuire L., Conley M., Johnson D.W., Ratanjee S.K. (2023). Re-Thinking Hyperkalaemia Management in Chronic Kidney Disease-Beyond Food Tables and Nutrition Myths: An Evidence-Based Practice Review. Nutrients.

[84] Ramos C.I., González-Ortiz A., Espinosa-Cuevas A., Avesani C.M., Carrero J.J., Cuppari L. (2021). Does dietary potassium intake associate with hyperkalemia in patients with chronic kidney disease?. Nephrol. Dial. Transpl..

[85] Gai W., Lin L., Wang Y., Bian J., Tao Y. (2024). Relationship between dietary fiber and all-cause mortality, cardiovascular mortality, and cardiovascular disease in patients with chronic kidney disease: A systematic review and meta-analysis. J. Nephrol..

[86] Stevens P.E., Ahmed S.B., Carrero J.J., Foster B., Francis A., Hall R.K., Herrington W.G., Hill G., Inker L.A., Kazancıoğlu R. (2024). KDIGO 2024 Clinical Practice Guideline for the Evaluation and Management of Chronic Kidney Disease. Kidney Int..

[87] Wathanavasin W., Cheungpasitporn W., Thongprayoon C., Fülöp T. (2025). Effects of Dietary Fiber Supplementation on Modulating Uremic Toxins and Inflammation in Chronic Kidney Disease Patients: A Systematic Review and Meta-Analysis of Randomized Controlled Trials. Toxins.

[88] Hung T.V., Suzuki T. (2018). Dietary Fermentable Fibers Attenuate Chronic Kidney Disease in Mice by Protecting the Intestinal Barrier. J. Nutr..

[89] Mithul Aravind S., Wichienchot S., Tsao R., Ramakrishnan S., Chakkaravarthi S. (2021). Role of dietary polyphenols on gut microbiota, their metabolites and health benefits. Food Res. Int..

[90] Li C., Chen X., Zha W., Fang S., Shen J., Li L., Jiang H., Tian P. (2025). Impact of gut microbiota in chronic kidney disease: Natural polyphenols as beneficial regulators. Ren. Fail..

[91] Bao N., Chen F., Dai D. (2019). The Regulation of Host Intestinal Microbiota by Polyphenols in the Development and Prevention of Chronic Kidney Disease. Front. Immunol..

[92] Alvarenga L., Cardozo L., Leal V.O., Kemp J.A., Saldanha J.F., Ribeiro-Alves M., Meireles T., Nakao L.S., Mafra D. (2022). Can Resveratrol Supplementation Reduce Uremic Toxin Plasma Levels From the Gut Microbiota in Nondialyzed Patients with Chronic Kidney Disease?. J. Ren. Nutr..

[93] Turki K., Charradi K., Boukhalfa H., Belhaj M., Limam F., Aouani E. (2016). Grape seed powder improves renal failure of chronic kidney disease patients. Excli J..

[94] Macena M.L., Nunes L., da Silva A.F., Pureza I., Praxedes D.R.S., Santos J.C.F., Bueno N.B. (2022). Effects of dietary polyphenols in the glycemic, renal, inflammatory, and oxidative stress biomarkers in diabetic nephropathy: A systematic review with meta-analysis of randomized controlled trials. Nutr. Rev..

[95] Gutiérrez O.M. (2021). Treatment of Iron Deficiency Anemia in CKD and End-Stage Kidney Disease. Kidney Int. Rep..

[96] Ribeiro M., Fonseca L., Anjos J.S., Capo-Chichi J.C.C., Borges N.A., Burrowes J., Mafra D. (2022). Oral iron supplementation in patients with chronic kidney disease: Can it be harmful to the gut microbiota?. Nutr. Clin. Pract..

[97] Xia Y., Luo Q., Huang C., Shi L., Jahangir A., Pan T., Wei X., He J., Liu W., Shi R. (2023). Ferric citrate-induced colonic mucosal damage associated with oxidative stress, inflammation responses, apoptosis, and the changes of gut microbial composition. Ecotoxicol. Environ. Saf..

[98] Liu H., Wu W., Luo Y. (2023). Oral and intravenous iron treatment alter the gut microbiome differentially in dialysis patients. Int. Urol. Nephrol..

[99] Ikizler T.A., Burrowes J.D., Byham-Gray L.D., Campbell K.L., Carrero J.J., Chan W., Fouque D., Friedman A.N., Ghaddar S., Goldstein-Fuchs D.J. (2020). KDOQI Clinical Practice Guideline for Nutrition in CKD: 2020 Update. Am. J. Kidney Dis..

[100] Wang A.Y., Elsurer Afsar R., Sussman-Dabach E.J., White J.A., MacLaughlin H., Ikizler T.A. (2024). Vitamin Supplement Use in Patients with CKD: Worth the Pill Burden?. Am. J. Kidney Dis..

[101] Zeb F., Osaili T., Hashim M., Alkalbani N., Papandreou D., Cheikh Ismail L., Naja F., Radwan H., Hasan H., Obaid R.S. (2025). Effect of Vitamin D Supplementation on Human Gut Microbiota: A Systematic Review of Randomized Controlled Trials. Nutr. Rev..

[102] Mo Y., Sun H., Zhang L., Geng W., Wang L., Zou C., Wu Y., Ji C., Liu X., Lu Z. (2021). Microbiome-Metabolomics Analysis Reveals the Protection Mechanism of α-Ketoacid on Adenine-Induced Chronic Kidney Disease in Rats. Front. Pharmacol..

[103] Di Iorio B.R., Rocchetti M.T., De Angelis M., Cosola C., Marzocco S., Di Micco L., di Bari I., Accetturo M., Vacca M., Gobbetti M. (2019). Nutritional Therapy Modulates Intestinal Microbiota and Reduces Serum Levels of Total and Free Indoxyl Sulfate and P-Cresyl Sulfate in Chronic Kidney Disease (Medika Study). J. Clin. Med..

[104] Rocchetti M.T., Di Iorio B.R., Vacca M., Cosola C., Marzocco S., di Bari I., Calabrese F.M., Ciarcia R., De Angelis M., Gesualdo L. (2021). Ketoanalogs’ Effects on Intestinal Microbiota Modulation and Uremic Toxins Serum Levels in Chronic Kidney Disease (Medika2 Study). J. Clin. Med..

[105] Baldo M.P., Serrano M.D.C., Pitzer Mutchler A., Lee Y. (2024). Editorial: Hold the salt: Dietary sodium’s effect on cardiovascular and kidney diseases. Front. Nutr..

[106] Wilck N., Matus M.G., Kearney S.M., Olesen S.W., Forslund K., Bartolomaeus H., Haase S., Mähler A., Balogh A., Markó L. (2017). Salt-responsive gut commensal modulates T(H)17 axis and disease. Nature.

[107] Hu L., Zhu S., Peng X., Li K., Peng W., Zhong Y., Kang C., Cao X., Liu Z., Zhao B. (2020). High Salt Elicits Brain Inflammation and Cognitive Dysfunction, Accompanied by Alternations in the Gut Microbiota and Decreased SCFA Production. J. Alzheimers Dis..

[108] Villela-Torres M.d.l.L., Prado-Uribe M.-d.-C., Díaz M.Á., Pablo H.Q., Soria-Castro E., Escofet N.E., Maldonado C.E.F., Paniagua R. (2024). Effect of High Sodium Intake on Gut Tight Junctions’ Structure and Permeability to Bacterial Toxins in a Rat Model of Chronic Kidney Disease. Arch. Med. Res..

[109] Wang X., Xu Y., Wang Y., Xu Y., Tian Y., Wang Y., Wang M. (2025). Poricoic Acid A Protects Against High-Salt-Diet Induced Renal Fibrosis by Modulating Gut Microbiota and SCFA Metabolism. Plant Foods Hum. Nutr..

[110] Chen L., He F.J., Dong Y., Huang Y., Wang C., Harshfield G.A., Zhu H. (2020). Modest Sodium Reduction Increases Circulating Short-Chain Fatty Acids in Untreated Hypertensives. Hypertension.

[111] Zhou C., Li Y., Huang M., Bai M., Xing Y. (2026). Mediterranean diet with high-phenolic EVOO slows kidney function decline and reduces inflammation in nondialysis CKD: A meta-analysis. Front. Nutr..

[112] Pérez-Torres A., Caverni-Muñoz A., González García E. (2022). Mediterranean Diet and Chronic Kidney Disease (CKD): A Practical Approach. Nutrients.

[113] D’Alessandro C., Giannese D., Piccoli G.B., Panichi V., Cupisti A. (2025). Ketogenic diets in chronic kidney disease patients: A review for skeptics by skeptics. J. Nephrol..

[114] Li W., Gong M., Wang Z., Pan H., Li Y., Zhang C. (2024). The gut microbiota changed by ketogenic diets contribute to glucose intolerance rather than lipid accumulation. Front. Endocrinol..

[115] Basciani S., Camajani E., Contini S., Persichetti A., Risi R., Bertoldi L., Strigari L., Prossomariti G., Watanabe M., Mariani S. (2020). Very-Low-Calorie Ketogenic Diets with Whey, Vegetable, or Animal Protein in Patients with Obesity: A Randomized Pilot Study. J. Clin. Endocrinol. Metab..

[116] Heinsen F.A., Fangmann D., Müller N., Schulte D.M., Rühlemann M.C., Türk K., Settgast U., Lieb W., Baines J.F., Schreiber S. (2016). Beneficial Effects of a Dietary Weight Loss Intervention on Human Gut Microbiome Diversity and Metabolism Are Not Sustained during Weight Maintenance. Obes. Facts.

[117] Torres J.A., Holznecht N., Asplund D.A., Amarlkhagva T., Kroes B.C., Rebello J., Agrawal S., Weimbs T. (2024). A combination of β-hydroxybutyrate and citrate ameliorates disease progression in a rat model of polycystic kidney disease. Am. J. Physiol. Ren. Physiol..

[118] Cukoski S., Lindemann C.H., Arjune S., Todorova P., Brecht T., Kühn A., Oehm S., Strubl S., Becker I., Kämmerer U. (2023). Feasibility and impact of ketogenic dietary interventions in polycystic kidney disease: KETO-ADPKD-a randomized controlled trial. Cell Rep. Med..

[119] Kalantar-Zadeh K., Joshi S., Schlueter R., Cooke J., Brown-Tortorici A., Donnelly M., Schulman S., Lau W.L., Rhee C.M., Streja E. (2020). Plant-Dominant Low-Protein Diet for Conservative Management of Chronic Kidney Disease. Nutrients.

[120] Sumida K., Lau W.L., Kovesdy C.P., Kalantar-Zadeh K., Kalantar-Zadeh K. (2021). Microbiome modulation as a novel therapeutic approach in chronic kidney disease. Curr. Opin. Nephrol. Hypertens..

[121] Zarantonello D., Brunori G. (2023). The Role of Plant-Based Diets in Preventing and Mitigating Chronic Kidney Disease: More Light than Shadows. J. Clin. Med..

[122] Stanford J., Stefoska-Needham A., Jiang X., McWhinney B., Hassan H.I.C., El-Omar E., Charlton K., Lambert K. (2025). High-Diversity Plant-Based Diet and Gut Microbiome, Plasma Metabolome, and Symptoms in Adults with CKD. Clin. J. Am. Soc. Nephrol..

[123] Sakaguchi Y., Kaimori J.-Y., Isaka Y. (2023). Plant-Dominant Low Protein Diet: A Potential Alternative Dietary Practice for Patients with Chronic Kidney Disease. Nutrients.

[124] Michail A., Andreou E. (2025). A Plant-Dominant Low-Protein Diet in Chronic Kidney Disease Management: A Narrative Review with Considerations for Cyprus. Nutrients.

[125] Kalantar-Zadeh K., Rhee C.M., Joshi S., Brown-Tortorici A., Kramer H.M. (2022). Medical nutrition therapy using plant-focused low-protein meal plans for management of chronic kidney disease in diabetes. Curr. Opin. Nephrol. Hypertens..

[126] Wu H.M., Sun H.J., Wang F., Yang M., Dong B.R., Liu G.J. (2014). Oral adsorbents for preventing or delaying the progression of chronic kidney disease. Cochrane Database Syst. Rev..

[127] Lu P.-H., Yu M.-C., Wei M.-J., Kuo K.-L. (2021). The Therapeutic Strategies for Uremic Toxins Control in Chronic Kidney Disease. Toxins.

[128] Su P.Y., Lee Y.H., Kuo L.N., Chen Y.C., Chen C., Kang Y.N., Chang E.H. (2021). Efficacy of AST-120 for Patients with Chronic Kidney Disease: A Network Meta-Analysis of Randomized Controlled Trials. Front. Pharmacol..

[129] Cha R.H., Kang S.W., Park C.W., Cha D.R., Na K.Y., Kim S.G., Yoon S.A., Kim S., Han S.Y., Park J.H. (2017). Sustained uremic toxin control improves renal and cardiovascular outcomes in patients with advanced renal dysfunction: Post-hoc analysis of the Kremezin Study against renal disease progression in Korea. Kidney Res. Clin. Pract..

[130] Sanaka T., Fujimoto K., Niwayama J., Nishimura H., Naito T., Higuchi C., Akizawa T., Koide K., Koshikawa S. (2003). Effect of combined treatment of oral sorbent with protein-restricted diet on change of reciprocal creatinine slope in patients with CRF. Am. J. Kidney Dis..

[131] Cooper T.E., Khalid R., Chan S., Craig J.C., Hawley C.M., Howell M., Johnson D.W., Jaure A., Teixeira-Pinto A., Wong G. (2023). Synbiotics, prebiotics and probiotics for people with chronic kidney disease. Cochrane Database Syst. Rev..

[132] Cedillo-Flores R., Cuevas-Budhart M.A., Cavero-Redondo I., Kappes M., Avila-Diaz M., Paniagua R. (2025). Impact of Gut Microbiome Modulation on Uremic Toxin Reduction in Chronic Kidney Disease: A Systematic Review and Network Meta-Analysis. Nutrients.

[133] Rossi M., Klein K., Johnson D.W., Campbell K.L. (2012). Pre-, pro-, and synbiotics: Do they have a role in reducing uremic toxins? A systematic review and meta-analysis. Int. J. Nephrol..

[134] Bian J., Liebert A., Bicknell B., Chen X.M., Huang C., Pollock C.A. (2022). Faecal Microbiota Transplantation and Chronic Kidney Disease. Nutrients.

[135] Barba C., Soulage C.O., Caggiano G., Glorieux G., Fouque D., Koppe L. (2020). Effects of Fecal Microbiota Transplantation on Composition in Mice with CKD. Toxins.

[136] Hu Z.B., Lu J., Chen P.P., Lu C.C., Zhang J.X., Li X.Q., Yuan B.Y., Huang S.J., Ruan X.Z., Liu B.C. (2020). Dysbiosis of intestinal microbiota mediates tubulointerstitial injury in diabetic nephropathy via the disruption of cholesterol homeostasis. Theranostics.

[137] Lu J., Chen P.P., Zhang J.X., Li X.Q., Wang G.H., Yuan B.Y., Huang S.J., Liu X.Q., Jiang T.T., Wang M.Y. (2021). GPR43 deficiency protects against podocyte insulin resistance in diabetic nephropathy through the restoration of AMPKalpha activity. Theranostics.

[138] Zhao J., Bai M., Yang X., Wang Y., Li R., Sun S. (2021). Alleviation of refractory IgA nephropathy by intensive fecal microbiota transplantation: The first case reports. Ren. Fail..

[139] Zhou G., Zeng J., Peng L., Wang L., Zheng W., Di W., Yang Y. (2021). Fecal microbiota transplantation for membranous nephropathy. CEN Case Rep..

[140] Paramsothy S., Kamm M.A., Kaakoush N.O., Walsh A.J., van den Bogaerde J., Samuel D., Leong R.W.L., Connor S., Ng W., Paramsothy R. (2017). Multidonor intensive faecal microbiota transplantation for active ulcerative colitis: A randomised placebo-controlled trial. Lancet.

[141] Arteaga-Muller G.Y., Flores-Treviño S., Bocanegra-Ibarias P., Robles-Espino D., Garza-González E., Fabela-Valdez G.C., Camacho-Ortiz A. (2024). Changes in the Progression of Chronic Kidney Disease in Patients Undergoing Fecal Microbiota Transplantation. Nutrients.

[142] Monda V., Villano I., Messina A., Valenzano A., Esposito T., Moscatelli F., Viggiano A., Cibelli G., Chieffi S., Monda M. (2017). Exercise Modifies the Gut Microbiota with Positive Health Effects. Oxid. Med. Cell. Longev..

[143] Mailing L.J., Allen J.M., Buford T.W., Fields C.J., Woods J.A. (2019). Exercise and the Gut Microbiome: A Review of the Evidence, Potential Mechanisms, and Implications for Human Health. Exerc. Sport. Sci. Rev..

[144] Min L., Ablitip A., Wang R., Luciana T., Wei M., Ma X. (2024). Effects of Exercise on Gut Microbiota of Adults: A Systematic Review and Meta-Analysis. Nutrients.

[145] Estaki M., Pither J., Baumeister P., Little J.P., Gill S.K., Ghosh S., Ahmadi-Vand Z., Marsden K.R., Gibson D.L. (2016). Cardiorespiratory fitness as a predictor of intestinal microbial diversity and distinct metagenomic functions. Microbiome.

[146] Chen J., Guo Y., Gui Y., Xu D. (2018). Physical exercise, gut, gut microbiota, and atherosclerotic cardiovascular diseases. Lipids Health Dis..

[147] de Brito J.S., Vargas D., da Silva G.S., Marinho S., Borges N.A., Cardozo L.F.M.F., Fonseca L., Ribeiro M., Chermut T.R., Moura M. (2022). Uremic toxins levels from the gut microbiota seem not to be altered by physical exercise in hemodialysis patients. Int. Urol. Nephrol..

